# Etiology and Visual Prognosis in Open Globe Injuries: Results of A Tertiary Referral Center in Turkey

**DOI:** 10.1038/s41598-019-54598-w

**Published:** 2019-11-29

**Authors:** Taylan Ozturk, Golgem Cetin Dora, Ziya Ayhan, Mahmut Kaya, Gul Arikan, Aylin Yaman

**Affiliations:** Department of Ophthalmology, Dokuz Eylul University School of Medicine, Izmir, Turkey

**Keywords:** Eye diseases, Trauma

## Abstract

We aimed to analyse the clinical characteristics of OGI and evaluate the correlation between baseline ocular trauma score (OTS) and visual outcomes in cases with OGI. The charts of 257 OGI patients who had at least six months of follow-up were reviewed retrospectively. Demographics, data about the etiology, localization and size of the OGI, baseline and final best-corrected visual acuity (BCVA) were noted. At the time of approval OTS was calculated and compared with final BCVA. All analysis was performed in both entire study population and our pediatric subgroup. A total of 261 eyes of 257 patients with a mean age of 34.9 ± 19.8 years were enrolled. Globe injury with a mean size of 6.7 ± 4.5 mm was within zone I in 46.7% of the eyes. Older age (p < 0.001, OR = 1.029, 95% CI = 1.015–1.043), higher baseline logMAR BCVA scores (p < 0.001, OR = 4.460, 95% CI = 2.815–7.065), bigger wound size (p < 0.001, OR = 1.159, 95% CI = 1.084–1.240), relative afferent pupillary defect (RAPD) positiveness (p < 0.001, OR = 0.021 95% CI = 0.005–0.087), lower OTS (p < 0.001, OR = 27.034, 95% CI = 6.299–116.021), presence of concomitant retinal detachment (p < 0.001, OR = 0.157, 95% CI = 0.080–0.306), and endophthalmitis (p = 0.045, OR = 0.207, 95% CI = 0.044–0.962) were found to be related to poor visual prognosis. Cases with OGI caused by a sharp object (p = 0.007, OR = 0.204, 95% CI = 0.065–0.641) and those injured by a glass (p = 0.039, OR = 0.229, 95% CI = 0.056–0.931) had more favorable final vision. This study highlights that baseline BCVA, wound size, RAPD, retinal detachment, and OTS were the most significant markers for poor visual outcomes in both the entire population and pediatric subgroup. In cases with OGI, OTS was also found effective in predicting visual prognosis.

## Introduction

Ocular trauma is one of the most important causes of avoidable vision loss all over the world, and constitutes 10–15% of all ophthalmologic diseases^1–8^. The World Health Organization (WHO) program for prevention of blindness estimated that annually there were 55 million people suffering from an eye injury requiring limitation in daily activities for more than 24 hours; 750000 of those were hospitalized, and 200000 cases had the diagnosis of open eye injuries. Owing to ocular trauma, unilateral visual loss has been diagnosed in approximately 19 million people, whereas bilateral blindness has occurred in 1.6 million cases globally^1^.

Birmingham Eye Trauma Terminology (BETT) system provides clinicians with clear definitions of widely used ocular trauma terminology. Ocular trauma is divided into closed wounds and open eye injury; which is defined as full-thickness damage of the cornea, sclera, or both^9^. Open globe injury (OGI) is an ophthalmologic emergency that requires early recognition and perfect surgical repair. Poorer baseline visual acuity, more serious damage to the posterior segment of the eye, and positive afferent pupillary defect are allied with higher rates of permanent visual loss. For predicting the visual outcomes at the end of sixth postoperative month in cases with traumatic eye injury, Kuhn *et al*.^10^ firstly suggested the calculation of ocular trauma score (OTS) that might guide clinicians in counseling and treating of such patients. In the present study, we evaluated the trauma etiology and profile of open eye injuries commonly seen in Turkish patients to find out the prognostic factors affecting the visual prognosis. Furthermore, the correlation between OTS and visual outcomes was analyzed in patients with OGI who underwent surgical intervention in our tertiary referral center. In order to develop an effective approach for decreasing the frequency of OGI that constitutes one of the most common causes of preventable blindness in working population, the need of current information about the mechanisms and causes of OGI is undisputed for a public health and injury prevention perspective.

## Materials and Methods

**Figure 1:**
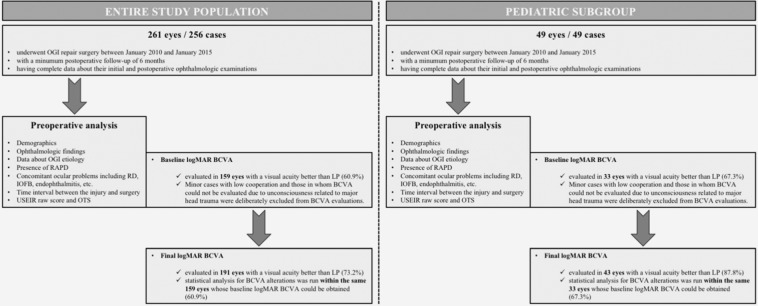
Definition of the study population.

The charts of 257 patients underwent surgical intervention for OGI repair in our center between January 2010 and January 2015 were reviewed retrospectively. Cases with a minimum postoperative follow-up of six months, and those had complete data about their initial and postoperative ophthalmologic examinations including visual acuity assessment, slit-lamp biomicroscopic and fundoscopic evaluation with B-scan ultrasonography and orbital computed tomography scans were included in the present study (Fig. 1). As the feature of our tertiary referral center that locates in western region of the country and attends a hinterland with higher socioeconomic and sociocultural status comparing with the remaining four major medical centers situated in our region, our study population consisted of urban rather than rural residents. A signed informed consent was obtained from all adult patients; however, parents or first-degree relatives of pediatric cases and unconscious subjects gave a written informed consent prior to any surgical interventions. The study was conducted in accordance with the methods described adhered to tenets of the Declaration of Helsinki, and the study protocol was approved by the Institutional Ethics Committee (Dokuz Eylul University Ethics Committee – Approval date and number: 30.07.2015 and 2015/18–16). This is a retrospective, non-comparative interventional case series, and all of the patients consented to our review of their medical records.

Patient demographics as well as data about the etiology, localization and size of the OGI, baseline and final best-corrected visual acuity (BCVA), the presence of a relative afferent pupillary defect (RAPD), concomitant ocular problems including retinal detachment (RD), intraocular foreign body (IOFB) and endophthalmitis diagnosed before the surgical repair, duration of the follow-up, and time interval between injury and surgical repair were noted for each participant. Regarding to the ocular findings of the study eyes at the time of approval, OTS was calculated retrospectively according to a calculation chart that was firstly reported by Kuhn *et al*.^10^, and used for estimating the probability of follow-up visual acuity range (Table 1). Minor cases with low cooperation and those in whom BCVA could not be evaluated due to unconsciousness related to major head trauma were deliberately excluded from visual acuity evaluations. For the purpose of analysis, visual acuity of more than hand motions was converted to logMAR unit according to the published manuscript of Holladay JT^11^. Study eyes were also grouped according to their OTS, and the frequency of good visual outcome defined as the final Snellen BCVA of being ≥20/40 was also calculated for each group.

**Table 1:** Ocular Trauma Score (OTS) calculation chart (Kuhn *et al*.^10^).

Variables	Raw points
Initial Visual Acuity	
NLP	60
LP/HM	70
1/200–19/200	80
20/200–20/50	90
≥20/40	100
Rupture	−23
Endophthalmitis	−17
Perforating injury	−14
Retinal detachment	−11
RAPD	−10

**NLP:** No light perception/**LP:** Light perception/**HM:** Hand motions/**RAPD:** Relative afferent pupillary defect.

Primary repair was the treatment of choice for the first operation in all eyes except for those with an anterior capsular rupture of crystalline lens and vitreous prolapse into the anterior chamber. Such eyes underwent primary repair combined with lensectomy and anterior vitrectomy (if needed) but any intraocular lens (IOL) implantation was not performed in the first operation. Aphakic cases with favorable visual prognosis underwent IOL implantation within three months after primary repair. Final visual outcomes were also evaluated with respect of the time between eye injury and surgical intervention. The localization of OGI was defined according to BETT as being in zone I if the injury concerns cornea and limbus; zone II if the injury involves the sclera within the 5 mm from the limbus; and zone III if it places more than 5 mm from the limbus. The study population was divided into three subgroups according to this localization terminology, and visual prognosis was compared among these subgroups. Total number of surgical interventions for each case was also reported in such subgroups.

The data were stored on a computerized database and analyzed using SPSS version 22.0 statistical software (IBM, Armonk, NY). After normality was tested with Kolmogorov-Smirnov test, Mann-Whitney U test, Wilcoxon signed ranks test, and Spearman correlation tests were used for statistical analysis where appropriate. A BCVA level of <20/200 was defined as poor vision according to WHO criteria, and a multivariable logistic regression analysis was performed to find out the relation between poor final visual prognosis and those variables found to be significantly associated with the visual outcomes in the univariable analysis. A probability value of below 0.05 was considered as significant. Fifteen years of age was considered as the cut-off for defining pediatric subgroup, and all statistical analyses were also performed in our pediatric population.

## Results

**Figure 2:**
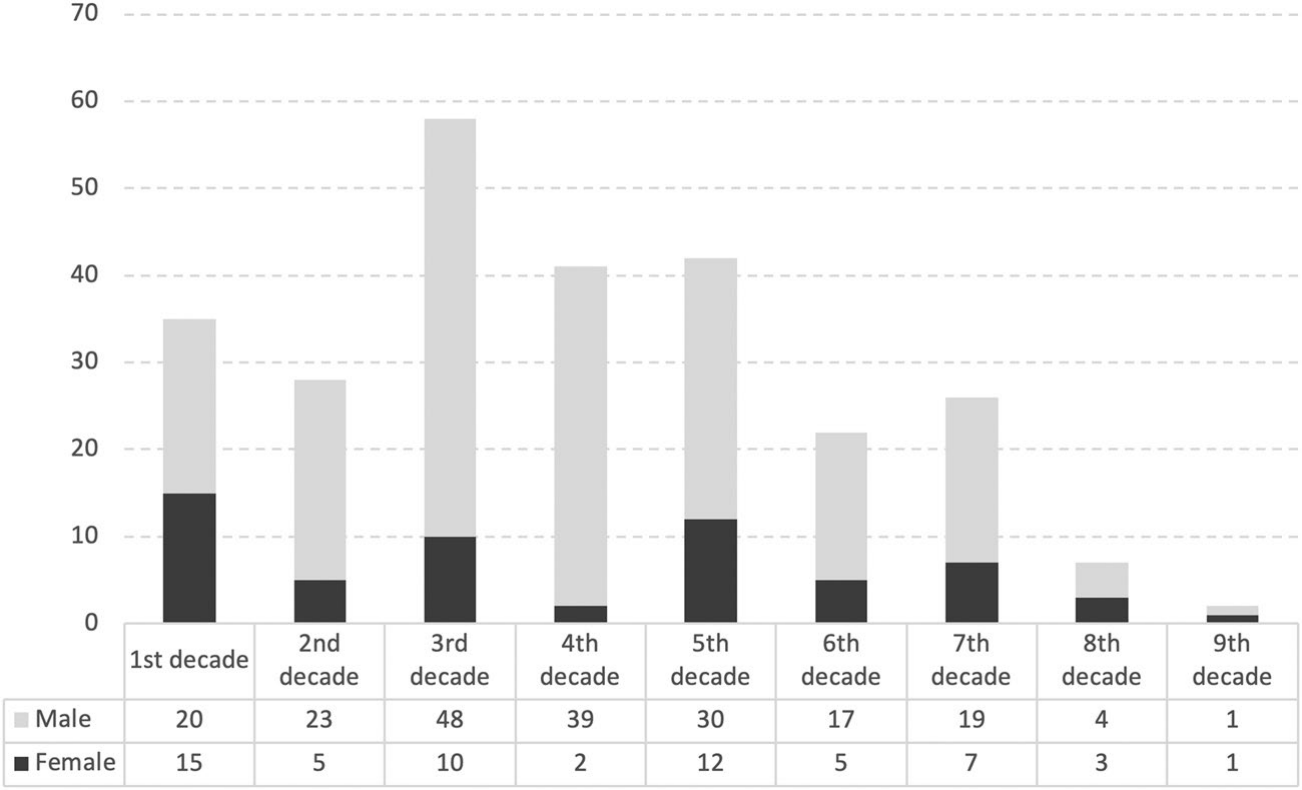
Male to female ratio of study population.

Of the 257 patients, 59 were female (23.0%) and 198 were male (77.0%). There was a preponderance of males in the entire study population (male to female ratio of 3.3/1), and the relative ratio of males was the highest (19.5/1) in the fourth decade (Fig. 2). Mean age was found as 34.9 ± 19.8 years (range, 1–85 years) at the time of eye injury, and 19.1% comprised of pediatric cases. Four patients (1.6%) were diagnosed with bilateral OGI, hence a total of 261 eyes were enrolled in this study. Work related injuries accounted for 29.1% of all OGI, followed by injuries occurred at home (22.2%), in road traffic accidents (14.5%), and during daily sport activities including contact sports accidents and personal injuries occurred with leg stretching bands, pilates resistance cords or a shuttlecock (9.2%). After minor cases with low cooperation and those in whom BCVA could not be evaluated due to unconsciousness related to major head trauma were deliberately excluded, baseline BCVA was found as a mean of 1.76 ± 1.22 logMAR in 159 eyes (60.9%) with a visual acuity better than light perception (LP). Mean BCVA was 0.87 ± 0.99 logMAR in such 159 eyes at the last follow-up visit (p < 0.001). However, BCVA assessment could be evaluated in a total of 191 eyes (73.2%) with a visual acuity better than LP at the last visit, and mean final BCVA was 1.00 ± 1.04 logMAR among them. Final Snellen BCVA of ≥ 20/40 was achieved in 3.5%, 22.0%, and 75.4% of the eyes with OTS 1, 2, and 3, respectively. Surgical intervention within the first 6 hours of OGI was performed in 10.0% of the cases, whereas those operated within the first 24 hours of ocular trauma constituted 72.8% of the study eyes. Period between injury and repair time was not found to be related with final visual outcomes in our study population (p = 0.647, r = 0.033). Size of globe injury was found to be negatively correlated with raw trauma scores (p < 0.001, r = −0.344) and positively correlated with baseline and final logMAR BCVA scores (p = 0.001, r = 0.251 and p < 0.001, r = 0.274, respectively). Mean number of surgical intervention was found as 1.48 ± 0.67 (range, 1–4). Phthisis bulbi was diagnosed in 5 eyes after a mean follow-up of 15.7 ± 19.2 months (range, 6–60 months). Demographics and ocular findings with their effects over final BCVA were given in Table 2. Older age (p < 0.001, r = 0.263), higher baseline logMAR BCVA scores (p < 0.001, r = 0.636), bigger wound size (p < 0.001, r = 0.276), lower OTS (p < 0.001), having a shotgun injury (p = 0.035), as well as presence of RAPD (p < 0.001), RD (p < 0.001), and endophthalmitis (p = 0.038) were found to be related with increased logMAR BCVA scores at the last control visit.

**Table 2:** Demographics and ocular findings with their effects over final logMAR BCVA in cases with open globe injury (OGI).

	Entire study population		Pediatric population	
	n = 261 eyes	p value	n = 49 eyes	p value
Gender (n; %)		0.472^**†**^		0.784^**†**^
Female	59; 23.0%		17; 34.7%	
Male	198; 77.0%		32; 65.3%	
Age (years)	34.9 ± 19.8	<0.001^**‡**^	8.0 ± 4.5	0.552^**‡**^
Bilateral involvement (n; %)	4; 1.6%		—	
Wound localization (n; %)		0.886^**+**^		0.251^**+**^
Zone I	121; 46.4%		26; 53.1%	
Zone II	70; 26.8%		9; 18.4%	
Zone III	70; 26.8%		14; 28.6%	
Wound size (mm)	6.7 ± 4.5	<0.001^**‡**^	7.1 ± 4.9	0.047^**‡**^
Nature of OGI (n; %)		0.035^**+**^		0.257^**+**^
Sharp objects	139; 53.3%		33; 67.3%	
Blunt trauma	100; 38.3%		15; 30.6%	
Shot-gun missile	22; 8.4%		1; 2.0%	
Agents causing OGI (n; %)		0.097^**+**^		0.196^**+**^
Metal	109; 41.8%		21; 42.9%	
Wood/Thorn	54; 20.7%		6; 12.2%	
Glass	47; 18.0%		12; 24.5%	
Stone	21; 8.0%		—	
Fist blow/Fingernail	14; 5.4%		3; 6.1%	
Plastic	14; 5.4%		5; 10.2%	
Farm animal	2; 0.8%		2; 4.1%	
Concomitant ocular problems				
Relative afferent pupillary defect (RAPD)	62; 23.8%	<0.001^**†**^	10; 20.4%	0.003^**†**^
Iris/choroidal tissue prolapse	119; 45.6%	0.921^**†**^	28; 57.1%	0.564^**†**^
Retinal detachment (RD)	73; 28.0%	<0.001^**†**^	6; 12.2%	0.017^**†**^
Intraocular foreign body (IOFB)	58; 22.2%	0.558^**†**^	7; 14.3%	0.185^**†**^
Endophthalmitis	12; 4.6%	0.038^**†**^	—	
Preoperative BCVA (logMAR)	1.76 ± 1.22^*^	<0.001^**‡**^	1.71 ± 1.02^**^	0.003^**‡**^
Preoperative BCVA (Snellen)		<0.001^**+**^		0.010^**+**^
NLP	27; 10.3%		3; 6.1%	
LP/HM	144; 55.2%		22; 44.9%	
1/200–19/200	31; 11.9%		14; 28.6%	
20/200–20/50	24; 9.2%		6; 12.2%	
≥20/40	35; 13.4%		4; 8.2%	
Raw score	49.5 ± 14.8	<0.001^**‡**^	53.4 ± 11.8	0.001^**‡**^
OTS		<0.001^**+**^		0.009^**+**^
1	86; 33.0%		8; 16.3%	
2	118; 45.2%		30; 61.2%	
3	57; 21.8%		11; 22.4%	
4	—		—	
5	—		—	
Mean OTS	1.89 ± 0.73		2.04 ± 0.61	
Operation time (h)				
Within the first 6 hours of OGI (n; %)	26; 10.0%	0.770^**†**^	7; 14.3%	0.400^**†**^
Within the first day of OGI (n; %)	190; 72.8%	0.701^**†**^	38; 77.6%	0.794^**†**^
Total number of surgical intervention	1.48 ± 0.67	0.073^**+**^	1.63 ± 0.64	0.314^**+**^

**BCVA:** Best-corrected visual acuity**/NLP:** No light perception**/LP:** Light perception**/HM:** Hand motions**/OTS:** Ocular trauma score**/**^**†**^Mann Whitney U test was used**/**^**‡**^Spearman correlation analysis was used**/**^**+**^Kruskal-Wallis test was used/^*^Preoperative BCVA was obtained from 159 eyes/^**^Preoperative BCVA was obtained from 33 eyes.

After performing a univariable logistic regression analysis, factors that were found to be related with poor visual outcomes defined as a final BCVA of < 20/200 were included in a multivariable logistic regression analysis for further evaluation. Older age (p < 0.001, OR = 1.029, 95% CI = 1.015–1.043), higher baseline logMAR BCVA scores (p < 0.001, OR = 4.460, 95% CI = 2.815–7.065), bigger wound size (p < 0.001, OR = 1.159, 95% CI = 1.084–1.240), positive RAPD (p < 0.001, OR = 0.021 95% CI = 0.005–0.087), lower OTS (p < 0.001, OR = 27.034, 95% CI = 6.299–116.021), presence of concomitant RD (p < 0.001, OR = 0.157, 95% CI = 0.080–0.306), and endophthalmitis (p = 0.045, OR = 0.207, 95% CI = 0.044–0.962) were found to be the most significant parameters related to poor visual prognosis. Cases with OGI caused by a sharp object (p = 0.007, OR = 0.204, 95% CI = 0.065–0.641) and those traumatized by a glass (p = 0.039, OR = 0.229, 95% CI = 0.056–0.931) had more favorable final vision (Table 3).

**Table 3:** Multivariable logistic regression analysis of factors that affected final visual outcomes.

	Entire study population (261 eyes/257 cases)			Pediatric population (49 eyes/49 cases)		
	p value	OR	95% CI	p value	OR	95% CI
Gender	0.796			0.246		
Age	<0.001^*^	1.029	1.015–1.043	0.093		
Interval between OGI and surgery	0.791			0.443		
Operation within first 6 h of OGI	0.507			0.899		
Operation within first day of OGI	0.801			0.785		
Zone of injury	0.278			0.032^*^	0.174	0.035–0.864
Wound size*	<0.001^*^	1.159	1.084–1.240	0.015^*^	1.177	1.032–1.343
Nature of OGI	0.007^*^	0.204	0.065–0.641	0.913		
Agents causing OGI	0.039^*^	0.229	0.056–0.931	0.043^*^	0.061	0.004–0.919
RAPD	<0.001^*^	0.021	0.005–0.087	0.001^*^	0.020	0.002–0.150
Iris/Choroidal tissue prolapse	0.281			0.135		
Retinal detachment (RD)	<0.001^*^	0.157	0.080–0.306	0.015^*^	0.061	0.006–0.581
Intraocular foreign body (IOFB)	0.622			0.115		
Endophthalmitis	0.045^*^	0.207	0.044–0.962	—		
Baseline logMAR BCVA	<0.001^*^	4.460	2.815–7.065	0.012^*^	3.761	0.958–14.774
Raw score	<0.001^*^	0.867	0.836–0.899	0.004^*^	0.889	0.820–0.963
Ocular Trauma Score (OTS)	<0.001^*^	27.034	6.299–116.021	0.005^*^	70.000	3.718–1317.868

**OGI:** Open globe injury**/RAPD:** Relative afferent pupillary defect**/BCVA:** Best-corrected visual acuity/^*^A multivariable logistic regression analysis was performed on factors found significant in univariable logistic regression analysis in order to identify their association with poor visual outcomes.

A total 49 eyes of 49 children constituted our pediatric subgroup, and data about their demographics and ocular findings were summarized in Table 2. On the day of admission, a visual acuity better than LP could be evaluated in 33 children (67.3%) who had a mean BCVA of 1.71 ± 1.02 logMAR, whereas final BCVA was found as a mean of 0.43 ± 0.53 logMAR among them (p < 0.001). In our pediatric subgroup, BCVA assessment could be evaluated in a total of 43 cases (87.8%) with a visual acuity better than LP at the last visit, and mean final BCVA was 0.57 ± 0.62 logMAR in such cases. Final Snellen BCVA of ≥ 20/40 was achieved in 12.5%, 40.0%, and 72.7% of the pediatric eyes with OTS 1, 2, and 3, respectively. Although, there was no statistical significance between final BCVA scores and zonal OGI localization (p = 0.251); pediatric eyes with zone III injury were found to be more prone to poor visual prognosis than those with zone I and II injury. Mean interval between the ocular injury and primary repair was 20.2 ± 18.7 hours (range, 4 hours–4 days), and no significant correlation was found between such interval and final logMAR BCVA scores in our pediatric population (p = 0.138, r = 0.230). Multivariable logistic regression analysis revealed that initial logMAR visual acuity (p = 0.012, OR = 3.761, 95% CI = 0.958–14.774), bigger wound size (p = 0.015, OR = 1.177, 95% CI = 1.032–1.343), positive RAPD (p = 0.001, OR = 0.020 95% CI = 0.002–0.150), presence of concomitant RD (p = 0.015, OR = 0.061, 95% CI = 0.006–0.581), and OTS (p = 0.005, OR = 70.000, 95% CI = 3.718–1317.868) were the most significant risk factors for poor visual outcomes in our pediatric subgroup. Furthermore, prognosis was better in children with both zone I OGI (p = 0.032, OR = 0.174, 95% CI = 0.035–0.864) and glass injury (p = 0.043, OR = 0.061, 95% CI = 0.004–0.919) (Table 3).

## Discussion

Open globe injuries are estimated to occur with a worldwide annual incidence of 3.5/100000, and have been defined as preventable cause of permanent visual impairment^1–8^. While advances in ophthalmic surgery techniques, instrumentations, and postoperative visual rehabilitation programs provide decreased blindness risk, OGI still constitutes one of the major causes of visual morbidity, and burdens a significant socioeconomic impact over society^1–5,12–14^. Although, statistics indicate that patients who have trauma in one eye are likely to have trauma in the fellow eye later in life, simultaneous trauma of both eyes is not so common. Cillino *et al*.^15^ reported only 8 bilateral cases after reviewing the charts of 152 patients with closed globe injury and 146 patients with OGI. Meng *et al*.^16^ indicated that the rate of bilateral involvement had been 5.4% among their study patients with OGI. Patients referred to our clinic with bilateral OGI also composed only 1.6% of the entire study population. Published literature about ocular injuries has described a bimodal incidence pattern with two peaks seen in second and third decades of life as well as older adults^15–18^. In accordance with the literature, 54.0% of our study population was between the third and fifth decades of their life. Studies have revealed a male preponderance for OGI especially more significant in the middle decades; which is also in accordance with our results^15–18^.

Knyazer *et al*.^5^ reported that 78% of OGI was caused by sharp objects in males, while blunt trauma with a frequency of 63.2% was most common among females. In our study OGI related to sharp objects was also found to be more frequent in males (55.7%), and OGI caused by a blunt ocular trauma was more common in females (46.7%). Concomitant intraocular foreign body (IOFB) was present in 22.2% of our study population; which is in accordance with the recent literature^5–7,15,16^. Body part, sport equipment, and work tool were reported as the most common etiologic factors for OGI-related visual impairment, and metal objects were found as the most frequent cause of OGI in several studies^5–7,14–17^. The most frequent agents related with OGI were metal (41.8%), wood or thorn (20.7%), and glass (18.0%) in the present study. Patients injured at work constituted approximately one third of our study population, so in high-risk works for an eye injury, eye protection ought to be an obligation that has to be encouraged by the government policies. Furthermore, most of the glass injuries occurred in road traffic accidents might be prevented by wearing seatbelts that would also be a vital advocacy issue. Airbags are also known to reduce the rate or severity of eye injuries in motor vehicle accidents. Besides, it would be helpful to enforce local and global companies on performing innovations on motor vehicle designs according to the recent results of their safety and crush tests. Governments also ought to have strict regulations on vehicle certification in the course of importing and exporting new vehicles. In order to control its safety, every motor vehicle has to be inspected regularly by a legal institution; which has an authority to ban failed vehicles from traffic. However, such regulations are recently fashioned by the government in our country, old and low safety vehicles are still on the road especially in rural areas. Moreover, high percentage of accidents caused by drunk driving is another issue that has to be solved for our country. Public information and education campaigns are crucial to provide an awereness on avoiding from driving after consumption of any alcoholic drinks. Instead of ever-increasing penalties for drunk driving, alcohol detection devices that could be installed into the safety system of any motor vehicles may be much more effective as it can prevent the driver from starting the motor vehicle if he or she drinks alcohol more than the legal dose.

Various studies demonstrated that some of these risk factors are likely to predict poor visual prognosis after OGI including older age, poor initial BCVA, blunt trauma related injuries, zone II or III injuries, and lower OTS. Furthermore, OGI diagnosed eyes complicated with concomitant adnexal trauma, relative afferent pupillary defect, uveal tissue prolapse, hyphema, lens injury, RD, vitreous hemorrhage, and endophthalmitis have poor visual outcomes^5–10,15–21^. We also found that older age, lower initial BCVA, bigger wound size, positive RAPD, lower OTS, presence of concomitant RD and endophthalmitis were likely to be related with poor visual outcomes. Our results concur with studies that have shown the efficacy of OTS on predicting final visual outcome seen approximately six months after OGI^5,10,13–17,20,21^.

Over 70% of the cases with OGI were operated within the first day of trauma, and primary closure of the globe is commonly reported as the surgical method of choice^4–7,15–21^. Agrawal *et al*.^20^ published that 63% of their patients were managed with one surgery, whereas 27% required two and 10% required over three surgical interventions. Knyazer *et al*.^5^ reported a mean of 1.5 surgical intervention for their study group. Incidences of surgical intervention performed within the first 6 and 24 hours of OGI were 10.0% and 72.8%, respectively in our study. Mean number of total surgical intervention was also found as 1.48 ± 0.67. Although Du Toit *et al*.^21^ reported their overall evisceration rate as higher as 29.3% in their patients with OGI, advances in ophthalmic surgery techniques and instrumentations, as well as scheduling an early intervention have reduced the necessity of primary evisceration due to post-traumatic endophthalmitis, phthisis bulbi or sympathetic ophthalmia in such cases^3,5,8,12,16–18,22^. Occasional recovery of visual acuity was also published in cases with severe OGI and no light perception^22,23^. Since, we performed primary closure even in cases who could be estimated as a candidate for further evisceration surgery, and within our follow-up period phthisis bulbi was diagnosed in only 5 cases who underwent evisceration surgery just for cosmetic purpose.

Studies have demonstrated that most pediatric cases occur at home and are caused by sharp object penetration; which is in accordance with our results^24–27^. Our study demonstrated that home and school environment were the commonest places where OGI occurred in pupils. Since such eye injuries develop during unsupervised activities of the children, parents, teachers and caregivers should always be alerted on childcare especially at their playtimes. Children should also be educated about the risks of dangerous objects such as sharp tools, stones, sticks or thorns, and their caregivers ought to convince them to avoid from playing with high-risk objects. Factors related with poor visual outcomes have been reported similar to those seen in adult cases; which were young age, poor initial BCVA, presence of RAPD, the size and zone localization of injury as well as the presence of lens involvement, vitreous hemorrhage, RD, and endophthalmitis^24–30^. Zone I is the most common localization of OGI in children, whereas posteriorly placed lesions carry more risk for poor visual prognosis^24–29^. Similar to these results, zone I lesions that was found more prone to better visual outcomes, were also most common in our pediatric population. Since traumatic cataracts, corneal opacities secondary to scarring, band keratopathy or permanent staining of the cornea with blood, and against the rule corneal astigmatism secondary to corneal and limbal lacerations are the major causes of amblyopia in children with OGI, the management of such pathologies by a pediatric ophthalmologist following the primary closure surgery is crucial for better visual prognosis^24–27^. Poor initial visual acuity that may be associated with low cooperation level soon after an ocular trauma is common in children; however good visual prognosis defined as having a final Snellen BCVA of ≥ 20/40 is reported up to 63.4%^25–30^. In the present study 40.8% of our pediatric population also had a final BCVA of ≥ 20/40, and OTS was found as an effective tool to predict visual prognosis in Turkish children with OGI as well; which is also in accordance with recent literature^26–29^.

Our study has several limitations. First, the study design is retrospective and patients with poor visual recovery who lost to follow-up before the sixth postoperative month as well as severe multitrauma cases who died within the early postoperative weeks may likely to carry a selection bias. Secondly, the wide interval of age distribution may create a demographic bias; which was tried to overcome by evaluating the results of our pediatric population individually. Finally, the impact of many etiologic and ocular factors over the visual prognosis has been evaluated in the literature; however, there are various factors in such cases with head trauma that may affect visual acuity. We also deliberately excluded the data about visual acuity assessments in minor cases with low cooperation and those in whom BCVA could not be evaluated due to unconsciousness related to major head trauma. On the other hand, converting the visual acuity scores into logMAR system provided us to perform visual acuity analysis for the cases with a BCVA between hand motions and 20/400.

Demographic data and etiologic factors as well as final visual outcomes in Turkish population were found to be correlated with previously published reports about OGI from various countries. Age, wound size, initial BCVA score, OTS, positive RAPD, concomitant RD and endophthalmitis were the prognostic factors for poor visual prognosis; however, OGI caused by a sharp object and glass related traumas promised more favorable outcomes for our study population. OTS may help clinicians predict the prognosis of affected eyes. Prospective studies with large cohorts are still needed to clarify either prevention strategies or prognostic factors for better visual prognosis in cases with OGI. The majority of ocular injuries can be prevented by relatively simple measures such as wearing protective eyewear in risky workplaces or during contact sports. For children, adequate supervision and restriction of access to sharp objects must be strictly encouraged. Data obtained from similar studies would also be useful in developing educational material for the prevention of eye injuries and in advocacy at a government level for funding and programs to prevent injuries.
